# Editorial: Advances and challenges in stroke therapy: a regenerative prospective, volume II

**DOI:** 10.3389/fnhum.2026.1867182

**Published:** 2026-05-26

**Authors:** Syed Shadab Raza, Viola B. Morris, Hassan Azari

**Affiliations:** 1 Laboratory for Stem Cell and Restorative Neurology, Department of Biotechnology, Era's Lucknow Medical College and Hospital, Era University, Lucknow, India; 2 Division of Cardiology, Department of Medicine, Emory University School of Medicine, Atlanta, GA, United States; 3 School of Podiatric Medicine, Barry University, Miami Shores, FL, United States

**Keywords:** ischemic stroke, mitochondrial dysfunction, neuroinflammation, neurodegeneration NLRP3 inflammasome, regulated cell death

Ischemic stroke continues to be a primary cause of mortality and long-term disability globally, representing a significant clinical and socioeconomic challenge. Even though thrombolysis and mechanical thrombectomy are two new ways to improve reperfusion, they are still not very effective because of short time windows and the strange effects of cerebral ischemia/reperfusion injury. Thus, there is an immediate necessity to discover therapeutic approaches that transcend vascular recanalization and tackle the intricate molecular and cellular mechanisms involved in injury and recovery. The articles in this Research Topic, “*Advances and challenges in stroke therapy: a regenerative prospective, volume II*,” show that more and more people are realizing that ischemic stroke is a disorder that affects the whole body and is caused by a combination of mitochondrial dysfunction, neuroinflammation, metabolic imbalance, controlled cell death, and a lack of ability to regenerate.

She, Liu et al. demonstrate that mitochondrial dysfunction serves as a central driver of ischemic brain injury by disrupting cellular metabolism and redox homeostasis. Their findings show that ischemia/reperfusion-induced mitochondrial damage leads to excessive ROS production, calcium overload, and release of pro-apoptotic factors, thereby promoting neuronal death. Importantly, they further establish that mitochondria act as a convergence point for multiple regulated cell death pathways, including ferroptosis and PANoptosis, suggesting that mitochondrial preservation could simultaneously modulate diverse downstream death mechanisms.

Peng et al. identify cuproptosis as a novel copper-dependent form of regulated cell death closely associated with mitochondrial metabolism. Their work highlights that disruption of copper homeostasis, particularly copper accumulation, directly impairs mitochondrial function and triggers a distinct necrotic-like death pathway, thereby expanding the spectrum of mitochondria-driven cell death mechanisms in stroke pathology.

Yang et al. further establish that mitochondrial dysfunction acts upstream of NLRP3 inflammasome activation. They demonstrate that damaged mitochondria and misfolded proteins, including amyloid-β and α-synuclein, activate NLRP3 in microglia and astrocytes, thereby driving chronic neuroinflammation. This study underscores mitochondria as critical initiators of inflammasome-mediated neurodegeneration.

Liu et al., She, Shao et al., and Tang, Wen et al. collectively elucidate the protective role of Sirt1 as a key NAD^+^-dependent regulator in stroke. Their studies show that Sirt1 modulates oxidative stress, inflammation, apoptosis, and blood–brain barrier integrity through deacetylation of NF-κB and NLRP3 inflammasome components. By suppressing inflammasome activation and pyroptosis while enhancing mitochondrial function, Sirt1 emerges as a critical metabolic switch linking energy homeostasis with neuroprotection and recovery.

Tang, Liu et al. further demonstrate that Taohong Siwu decoction promotes neurological recovery by enhancing glycolysis and upregulating angiogenic factors, thereby supporting vascular regeneration. Complementing this, Hassanein et al. provide meta-analytical evidence that intravenous mesenchymal stem cell therapy is both safe and effective in improving functional recovery, reinforcing the therapeutic potential of regenerative strategies in stroke.

Yan et al. report that combined treatment with diterpene ginkgolide meglumine and edaravone is superior to edaravone monotherapy in acute ischemic stroke. Their meta-analysis reveals improved neurological outcomes, reduced oxidative stress, enhanced hemorheological parameters, and decreased neuron-specific enolase levels, supporting a multi-target therapeutic approach.

Zhan et al. demonstrate that Shuxuening injection, when used as an adjunct therapy, significantly improves clinical efficacy, neurological function, and daily living activities in stroke patients. Similarly, Wang et al. highlight the therapeutic potential of GuHong injection, emphasizing its multi-target anti-inflammatory, antioxidant, and neuroprotective properties.

Overall, the studies included in this Research Topic offer a comprehensive and holistic view of the pathophysiology and therapy of stroke. They collectively underscore the interplay between mitochondrial dysfunction, inflammasome activation, metabolic dysregulation, and regeneration, emphasizing the idea that these mechanisms are not isolated but rather form a dynamic interplay. This has significant implications for therapeutic development, as it underscores that successful therapies must simultaneously address both the mechanisms of disease progression and regenerative processes. This idea of a regenerative prospective in the therapy of stroke goes beyond the idea of incorporating stem cell and/or angiogenic therapies and instead suggests a more holistic approach that incorporates various processes. As the field progresses, it will be crucial in bridging the gap between basic science and clinical application in order to improve clinical outcomes in stroke.

